# Emerging Therapeutic Approaches to Combat COVID-19: Present Status and Future Perspectives

**DOI:** 10.3389/fmolb.2021.604447

**Published:** 2021-03-08

**Authors:** Karthik Vivekanandhan, Poornima Shanmugam, Hamed Barabadi, Vigneshwaran Arumugam, Dharun Daniel Raj Daniel Paul Raj, Manikandan Sivasubramanian, Subbaiya Ramasamy, Krishnan Anand, Pandi Boomi, Balakumar Chandrasekaran, Selvaraj Arokiyaraj, Muthupandian Saravanan

**Affiliations:** ^1^ Department of Biotechnology, K. S. Rangasamy College of Technology, Tiruchengode, Tamilnadu, India; ^2^ Department of Pharmaceutical Biotechnology, School of Pharmacy, Shahid Beheshti University of Medical Sciences, Tehran, Iran; ^3^ School of Biosciences and Technology, Vellore Institute of Technology, Vellore, India; ^4^ Department of Biotechnology, Maulana Abul Kalam Azad University of Technology, Haringhata, India; ^5^ Department of Biotechnology, Saveetha School of Engineering, Saveetha Institute of Medical and Technical Sciences (SIMATS), Chennai, India; ^6^ Department of Biological Sciences, School of Mathematics and Natural Sciences, The Copperbelt University, Riverside, Zambia; ^7^ Department of Chemical Pathology, School of Pathology, Faculty of Health Sciences and National Health Laboratory Service, University of the Free State, Bloemfontein, South Africa; ^8^ Department of Bioinformatics, Alagappa University, Karaikudi, India; ^9^ Faculty of Pharmacy, Philadelphia University, Amman, Jordan; ^10^ Department of Food Science and Biotechnology, Sejong University, Seoul, South Korea; ^11^ Department of Microbiology and Immunology, Division of Biomedical Science, School of Medicine, College of Health Science, Mekelle University, Mekelle, Ethiopia; ^12^ AMR and Nanomedicine Laboratory, Department of Pharmacology, Saveetha Dental College, Saveetha Institute of Medical and Technical Sciences (SIMATS), Chennai, India

**Keywords:** COVID-19, SARS-CoV-2, therapeutics, antiviral drugs, vaccines, nano-based approaches

## Abstract

Coronavirus disease (COVID-19) has emerged as a fast-paced epidemic in late 2019 which is disrupting life-saving immunization services. SARS-CoV-2 is a highly transmissible virus and an infectious disease that has caused fear among people across the world. The worldwide emergence and rapid expansion of SARS-CoV-2 emphasizes the need for exploring innovative therapeutic approaches to combat SARS-CoV-2. The efficacy of some antiviral drugs such as remdesivir, favipiravir, umifenovir, etc., are still tested against SARS-CoV-2. Additionally, there is a large global effort to develop vaccines for the protection against COVID-19. Because vaccines seem the best solution to control the pandemic but time is required for its development, pre-clinical/clinical trials, approval from FDA and scale-up. The nano-based approach is another promising approach to combat COVID-19 owing to unique physicochemical properties of nanomaterials. Peptide based vaccines emerged as promising vaccine candidates for SARS-CoV-2. The study emphasizes the current therapeutic approaches against SARS-CoV-2 and some of the potential candidates for SARS-CoV-2 treatment which are still under clinical studies for their effectiveness against SARS-CoV-2. Overall, it is of high importance to mention that clinical trials are necessary for confirming promising drug candidates and effective vaccines and the safety profile of the new components must be evaluated before translation of *in vitro* studies for implementation in clinical use.

## Introduction

The COVID-19 outbreak made the entire world frightened in late 2019, which is caused by severe acute respiratory syndrome coronavirus-2 (SARS-CoV-2) belongs to single stranded RNA viruses having spike-like projections of glycoprotein. The virus infection was first reported in Wuhan, People’s Republic of China during December 2019 (Chen et al., 2020). COVID-19 is the official name of the coronavirus declared by the World Health Organization (WHO). Even though the source of coronavirus has not been declared officially, bats and snakes are considered as the potential host. Wuhan institute of virology confirmed that 96% of similarity coronavirus with the gene sequence of bat coronavirus (Wang W. et al., 2020; Zhou P. et al., 2020). Coronavirus infects humans via the binding of S-protein with angiotensin-converting enzyme-2 (ACE-2) with higher affinity (Wrapp et al., 2020). Transmission of coronavirus is through respiratory droplets of infected persons. The common symptoms include fever, throat infection, cough, headache and breathlessness even some may be asymptomatic. The average incubation period of coronavirus ranges from three days to twenty-four days (Guan et al., 2020; Zhou et al., 2020a) but the prevalence is more in elderly people with medical comorbidities.

During this pandemic outbreak, several countries adopt preventive measures and their own treatment methodologies. Avoiding contact with infected persons, unnecessary travel and personal hygiene practices are the basic preventive measures followed to avoid the transmission of coronavirus. RT-PCR and chest computed tomography scan are the diagnostic tools used along with the combination of symptom relevant treatment (Velavan and Meyer, 2020). Viral infections are the major threat to the human kind. As of now, antiviral therapy, symptomatic and oxygen therapy are followed for treating SARS-CoV-2. Nano-based approaches are the promising tool for the diagnosis and treatment of such viral diseases. Based on the statistical analysis of StatNano, out of patents filed related to SARS-CoV-2 diagnostics and treatments, 5.2% belong to nano-based technology (Chakravarthy and Vora, 2020). With this background, the present review focuses on emerging approaches including drug repurposing, vaccine development including peptide and nano based approaches for COVID-19 therapeutics.

## Emerging Approaches for COVID-19 Therapeutics

### Drug Repurposing Approach for COVID-19 Treatment

**FIGURE 1:**
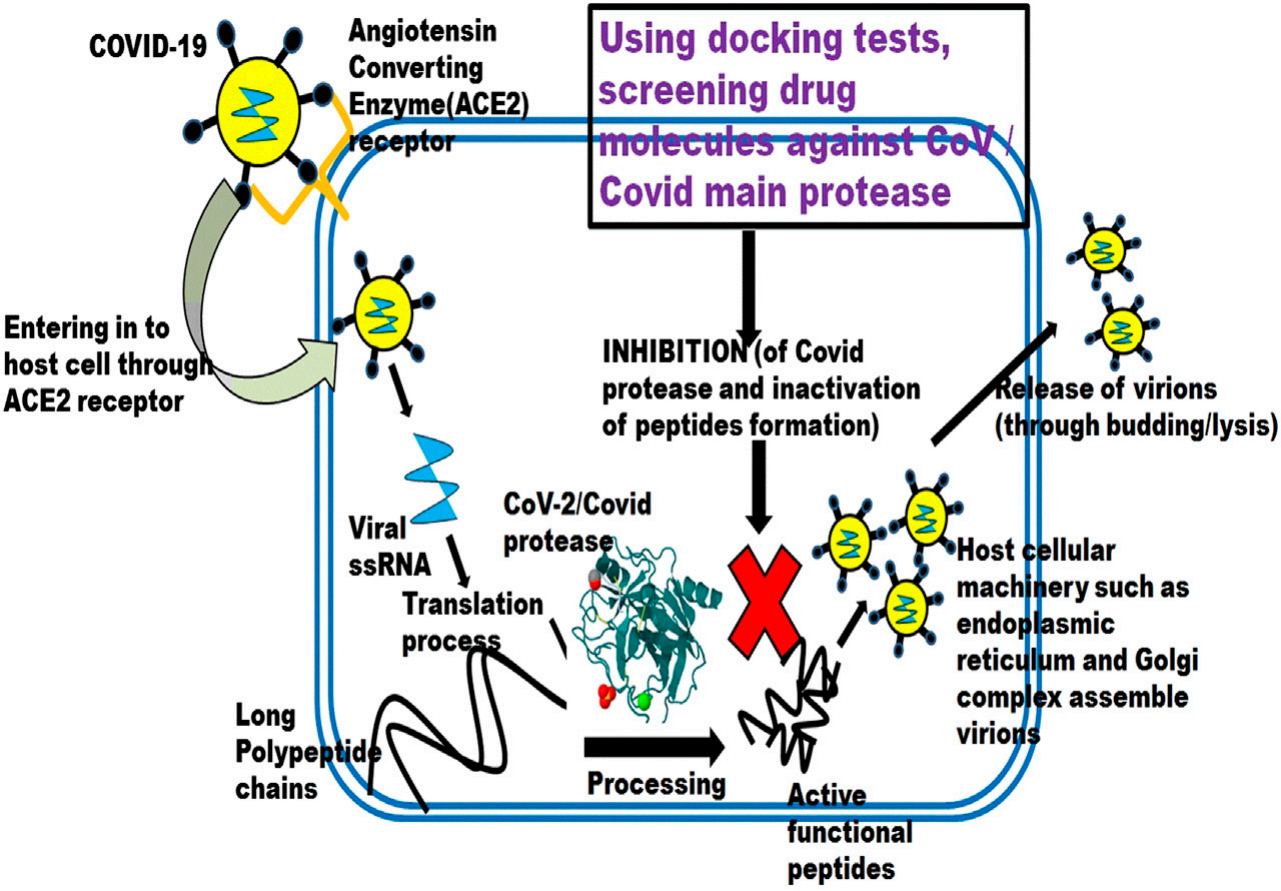
A schematic illustration of drug repurposing approach.

Even though a lot of potential antiviral drugs are available, their efficacy against SARS-CoV-2 is still tested for implementation. Below discussed drugs are some of the potential candidates for COVID-19 treatment and are under clinical study. Figure 1 depicts the schematic illustration of drug repurposing approach.

#### Remdesivir

Remdesivir, developed by Gilead Sciences (2009), is a broad-spectrum antiviral agent with a brand name of “veklury” administered as an intravenous injection. It was initially designed for Hepatitis C (Hep C) and respiratory syncytial virus (RSV). Later it was repurposed against Ebola and Marburg virus. Remdesivir has antiviral activity against filoviruses, pneumoviruses, paramyxoviruses, and coronaviruses *in vitro* (Lo and Jordan, 2017). Remdesivir, being an analogue of nucleotide, in its triphosphate form, i.e., Remdesivir triphosphate (RDV-TP), is used as a substrate for RNA dependent RNA polymerase, and has reported to inhibit the viral RNA synthesis by delayed termination of synthesis (Wang M. et al., 2020) in all corona viruses, including SARS-CoV-2. It was seen that RDV-TP resembles ATP (Adenosine triphosphate) (Saha et al., 2020). It competes with viral RNA synthesis, by forming a phosphodiester bond with the next nucleotide and terminates viral RNA formation at the third site from RDV-TP binding site, causing the termination of viral RNA synthesis in SARS-CoV-2 (Gordon et al., 2020).

Remdesivir exhibited an *in vitro* activity against SARS-CoV-2 in a preclinical study using *Rhesus macaque* model of SARS-CoV-2, the treatment was initiated soon after the *Rhesus macaque* was inoculated with SARS-CoV-2, and group of *Rhesus macaque* treated with remdesivir showed lower virus levels than untreated (Williamson et al., 2020). The toxicity and adverse effect of remdesivir is not clear yet and is to be investigated further (Fan et al., 2020). In clinical trials, remdesivir does not show gastrointestinal toxicity apart from minor diarrhoea in a few cases. In case of hepatotoxicity, elevations in the aminotransferases were noticed. In the case of nephrotoxicity, upon continual doses of remdesivir, reduced kidney function was observed. In case of respiratory toxicity, acute respiratory syndrome was observed in 4% of the patients treated with remdesivir.

The recommendation for the use of remdesivir arose from the multicentre, randomized placebo-controlled trials and the adaptive SARS-CoV-2 treatment trial (ACTT) (Wang M. et al., 2020). The study was conducted across 1603 SARS-CoV-2 infected patients. The patients in the trial group were affected to an extent that they require oxygen supplement, but mechanical ventilation (ECMO) is not required and the recovery period of 10–15 days was observed (Beigel et al., 2020). It is currently approved for treatment in the United States, India, Taiwan, Singapore and many other countries.

#### Hydroxychloroquine

Chloroquine is an anti-malarial drug developed in 1934. Later in 1946 hydroxychloroquine (HCQ), an analogue of chloroquine was developed to treat autoimmune diseases. HCQ has been used for the treatment of lupus, erythematosus, Q fever, certain types of malaria and rheumatoid arthritis (American society of health system pharmacist 2020). HCQ has fewer and less severe toxicities (including less propensity to prolong the QTc interval) and fewer drug-drug interactions than chloroquine. Studies show that HCQ increases the endosomal pH inhibiting fusion of SARS-CoV-2 with the host cell membrane (Wang M. et al., 2020). They possess an immunomodulatory effect and also block the transport of SARS-CoV-2 from early endosomes to endolysosomes, which may be required for the release of viral genome (Liu et al., 2020).

Most deaths in SARS-CoV-2 patients occurred due to cytokine storms. Cytokines play an important role in normal immune responses, but releasing large amounts in the body all at once can be harmful. A cytokine storm can occur as a result of an infection, autoimmune condition, or other diseases. It is observed that HCQ can reduce cytokine storms (Cao, 2020). A recent study by Tang et al., (2020b) reported that HCQ reduced clinical symptoms through anti-inflammatory properties and recovery of lymphopenia. But still safety, side effects and effectiveness of HCQ are under study. Further, the benefits and risks associated with HCQ depends on patient medical history (Juurlink 2020).

FDA has approved the use of 800 mg (HCQ) on the first day, followed by 400 mg for the next seven days for COVID-19 treatment (US FDA – Hydroxychloroquinone fact sheet for patients). Higher dosage leads to arrhythmia and sometimes eventual death (Borba et al., 2020). Patients with a history of renal and liver disorders should be treated with care using HCQ as it leads to nephrotoxicity and hepatotoxicity (Rismanbaf and Zarei, 2020).

#### Lopinavir/Ritonavir

Lopinavir/Ritonavir, under the brand name Kaletra^®^, belong to the protease inhibitor class which is especially used in the treatment of retroviruses which helps in SARS-CoV-2 treatment (Rismanbaf and Zarei, 2020). Although it is not curing HIV, it prevents secondary infections by decreasing the viral count, which are the characteristic symptoms of acquired immune-deficiency syndrome. Replication of SARS-CoV-2 depends on the cleavage of a poly protein into a RNA-dependent RNA polymerase and helicase, which helps in replicating the viral genome in the host cells (Zumla et al., 2016). For the cleavage of the poly protein, two proteases enzymes including 3-chymotrypsin-like protease (3CLpro) and papain-like protease (PLpro) are responsible, found in SARS-CoV-2 (Tahir ul Qamar et al., 2020). Thus, protease inhibitors like lopinavir and ritonavir reduce the viral count in the infected person’s body system and help them in recovery. In an *In vitro* study, it was found that lopinavir/ritonavir inhibited the protease 3CLpro (Liu and Wang, 2020; Liu et al., 2020).

In a clinical research study with a sample size of 199 patients, the patients were given Lopinavir 400/Ritonavir 100 mg orally, twice a day. It was observed that the group which was under liponavir/ritonavir treatment, recovered with a shorter intensive care time rather than the untreated group, and also the rate of mortality was less, though it was not statistically significant (Cao et al., 2020). Most clinical studies that surround lopinavir and ritonavir have a very small sample size, but it has been approved for treatment by National institute of Health (NIH) United States. The use of lopinavir/ritonavir is not recommended for patients suffering from porphyria, cardiac problems and cardiac conduction problems. Other side effects on usage are diarrhoea, which might persist for a week after usage. In some cases, diabetes mellitus, pancreatitis and hepatic problems have been reported as side effects due to the administering of lopinavir/ritonavir (Joint Formulary Committee, 2020).

#### Umifenovir

Umifenovir, developed in Russia and China has been used for infections caused by Influenza A, B and prophylaxis. The main mode of action by umifenovir is blocking the fusion of virus to the cell/endosome by interfering with the hydrogen bond network in the phospholipid (Villalain, 2010). *In vitro* study on the effect of Umifenovir against SARS-CoV-1 and SARS-CoV-2 revealed that Umifenovir combined with protease inhibitor (Liponavir/Ritonavir) exhibited higher negative conversion rates (Deng et al., 2020). But the latter is superior in terms of faster recovery (Chang et al., 2020).

Though Umifenovir did not exhibit severe adverse side effects, gastric problems including digestion, diarrhoea and nausea are reported (Huang et al., 2020). Further, there is no report on nephrotoxicity or hepatotoxicity. But some clinical studies have declared that umifenovir is not effective against SARS-CoV-2 treatment (Huang et al., 2020). Based on a clinical study conducted in Jinyintan Hospital, Wuhan province, it was revealed that Umifenovir neither increases the clearance rate of SARS-CoV-2 nor accelerates the recovery of patients in any way (Lian et al., 2020). The effect of umifenovir in SARS-CoV-2 treatment, still requires clinical investigations for further understanding.

#### Favipiravir

Favipiravir was first developed in 2014 by Fujifilm Toyama Chemical Co. Ltd., Japan, for the treatment of a novel influenza strain that was resistant to neuraminidase inhibitors. It is an analogue of guanine with a pyrazine carboxamide structure. Favipiravir enters the infected cell by endocytosis, and at the site it is transformed into favipiravir ribofuranosyl phosphate via phosphoribosylation and phosphorylation. After transformation, the prodrug destroys the conservative catalytic domain of RNA-dependent RNA polymerase (RdRp), interrupting the nucleotide incorporation process, thus interfering with the life cycle of the virus and hindering its replication within the host cell.

The RdRp of SARS-CoV-2 is 10X more active than any other viral RdRp faced until now (Shanon et al., 2020). Favipiravir is highly recommended as it inhibits viral RNA by sparing the native DNA of the host cell. Chang et al., (2020) conducted a clinical trial on the effectiveness of favipiravir and umifenovir against SARS-CoV-2. The study revealed that the recovery rate in favipiravir was higher than umifenovir. Table 1 shows the mode of action of potential antiviral drugs repurposed for COVID-19.

**TABLE 1:** Mode of action of potential antiviral drugs repurposed for COVID-19.

Drug	Mode of action	References
Remdesivir	Binds to the viral-RNA dependent RNA polymerase, inhibiting the replication of the virus by terminating transcription of viral-RNA	Wit, (2020)
Hydroxychloroquine	Increases the endosomal pH inhibiting the fusion of SARS-CoV-2 with the host cell membrane	Tang et al. (2020a)
Lopinavir/Ritonavir	Inhibits the protein 3CLpro, required for cleaving poly protein into RNA dependent RNA polymerase and helicase, helps in transcription of Viral RNA.	Cao et al. (2020)
Umifenovir	Blocks the fusion of virus to the cell/endosome by interfering with the hydrogen bond network in the phospholipid	Lian et al. (2020)
Favipiravir	Destroys the conservative catalytic domain of RNA-dependent RNA polymerase (RdRp), interrupting the nucleotide incorporation process, thus interfering with the life cycle of the virus	Shannon et al. (2020)

Treatment with favipiravir causes hyperuricemia. The major adverse effects on use of favipiravir is teratogenicity. Teratogenicity is the phenomenon where some agents (teratogens) cause major birth defects. Thus, the use of favipiravir of women at the child bearing age or pregnant ladies is highly inadvisable. Even men treated with favipiravir are advised to use contraceptive for a week minimum or until favipiravir is out of the system, to avoid any teratogenicity (Evaluation and Licensing Division, Pharmaceutical and Food Safety Bureau., 2011. Report on the Deliberation Results—Avigan.)

### Vaccine Development Approach

Vaccine development involves different strategies including live attenuated, or inactivated virus, virus-like particles or other protein-based approaches, viral vector–based vaccines or nucleic acid–based vaccines (Chauhan et al., 2020). In live attenuated vaccines, virulence of the virus is removed but viability is retained which in turn helps the immune system to develop memory cells (Badgett et al., 2002). Virus-like particles are molecules that closely resemble viruses which are synthesized by the expression of the viral structural protein. The synthesized molecules can assemble themselves to virus-like particles and help the body to boost immunity (Zeltins 2013). Viral vector-based vaccines and nucleic acid-based vaccines, incorporate the antibody expressing gene into the cell to produce necessary antibodies to acquire immunity against infections. Drug discovery and development for SARS-CoV-2 can be facilitated by artificial intelligence and other computational tools (Tang et al., 2020b; Lin et al., 2020; Zhou et al., 2020b). Adjuvants such as MF59, AS03, CpG are considered for COVID-19 treatment (Chauhan et al., 2020) to enhance the vaccine efficacy (Weinberger, 2018).

**FIGURE 2:**
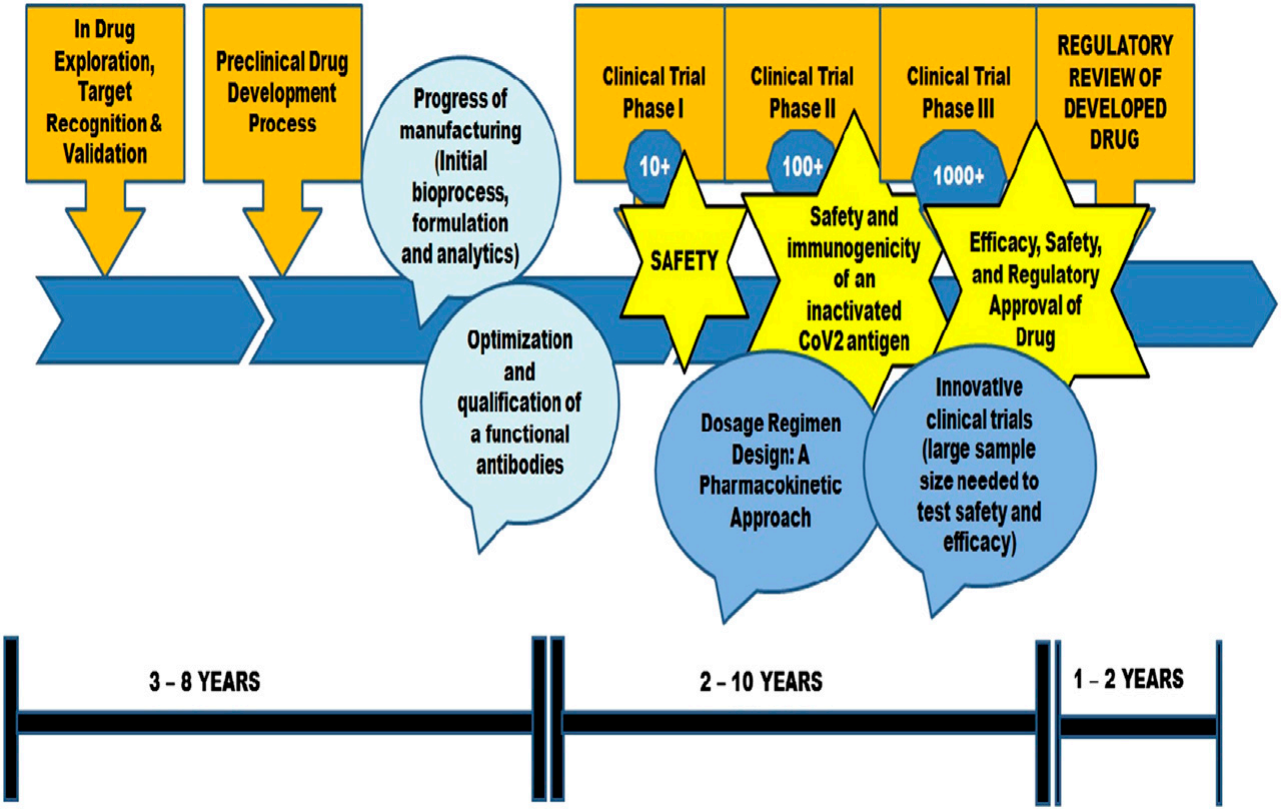
A schematic illustration of vaccine development approach.

Lipid nanoparticles (Adams et al., 2018), lipid coated mesoporous silica nanoparticles (LaBauve et al., 2018), Macrophage mimetic nanoparticles (MMNPs) (Zhang et al., 2020), Nano-Erythrocyte mimetic drug delivery (Cavezzi et al., 2020; Poduri et al., 2020), Nano-Platelet mimetic drug delivery (Anselmo et al., 2014), Nano-virus mimetic drug delivery (Ellah et al., 2020) have also been considered for COVID-19 therapeutics. The review includes discussion on peptide and nano based vaccine approaches for COVID-19 therapeutics. A complete list of vaccine candidates developed for COVID-19 is given in Supplementary Appendix (WHO-Draft landscape of COVID-19 candidate vaccines, 2021) (Table 4). Figure 2 depicts schematic illustration of vaccine development approach.

#### Peptide Based Vaccines

Peptide based vaccines are biologically safe and need not to be produced *in vitro*. Peptide vaccines outweigh the limitations of conventional vaccines by overcoming allergic reactions and autoimmune responses (Li et al., 2014). Peptide based vaccines are engineered to mimic the proteins or peptides in the pathogens, which can help in developing T-cells which are immunodominant (Malonis et al., 2020). Synthetic peptide vaccines are usually short amino acid sequences (20–30 amino acids in range) mimicking the specific epitope of the antigen of pathogen. B cells can recognize the mimicked peptide sequence and produce antibodies. T killer cells also kick in fast as the body responds by peptide based vaccine. Antibodies produced during infection target multiple antigen sites and over time some antibodies target specific antigen epitopes for immunity development. Bioinformatics tools facilitate finding the accessible peptide residue sites and a specific peptide can be engineered to bind the site. These tools help to narrow down to SARS-CoV-2-RBD (SARS-CoV-2-Receptor Binding Domain) to interact with hACE2 gaining entry to viral attachment and re-entry (Barh et al., 2020; Zhang et al., 2020).

Peptide derived from fermented soy cheese using *Lactobacillus delbrueckii* WS4 can be used as a potential antiviral agent for SARS CoV-2 (Chourasia et al., 2020). Peptide vaccine developed by IMV Inc., used DPX platform (Ye et al., 2020). Vaxil corporation developed signal peptide (Wu, 2020). Epivax and Generex Biotechnology Corporation proposed hybrid based Ii-Key peptide vaccine (Kallinters et al., 2020). Epivax proposed adjuvating peptide vaccines also (Wu, 2020). Table 2 shows the detailed list of peptide based vaccines developed for COVID-19 with platform description and developer.

**TABLE 2:** Peptide based vaccines developed for COVID-19.

Vaccine platform description	Developers
RBD protein delivered in mannose-conjugated chitosan nanoparticle	Ohio State University/Kazakh National Agrarian University
Recombinant spike protein with Essai O/W 1849101 adjuvant	Kazakh National Agrarian University
Peptides	Neo7Logic
Recombinant spike protein with Essai O/W 1849101 adjuvant	Kazakh National Agrarian University, Kazakhstan/National Scientific Center for Especially Dangerous Infections
Recombinant S protein	Max-Planck-Institute of Colloids and Interfaces
RBD protein (baculovirus production) + FAR-Squalene adjuvant	Farmacológicos Veterinarios SAC (FARVET SAC)/Universidad Peruana Cayetano Heredia (UPCH)
Protein Subunit	Research Institute for Biological Safety Problems, Rep of Kazakhstan
RBD-protein	Mynvax
Recombinant S protein	Izmir Biomedicine and Genome Center
Peptide + novel adjuvant	Bogazici University
S subunit intranasal liposomal formulation with GLA/3M052 adjs.	University of Virginia
S-Protein (Subunit) + Adjuvant, *E coli* based Expression	Helix Biogen Consult, Ogbomoso and Trinity Immonoefficient Laboratory, Ogbomoso, Oyo State, Nigeria
Protein Subunit S,N,M&S1 protein	National Research Centre, Egypt
Protein Subunit	University of San Martin and CONICET, Argentina
RBD protein fused with Fc of IgG + Adj.	Chulalongkorn University/GPO, Thailand
Capsid-like Particle	AdaptVac (PREVENT-nCoV consortium)
*Drosophila* S2 insect cell expression system VLPs	ExpreS2ion
Peptide antigens formulated in LNP	IMV Inc
S Protein	WRAIR/USAMRIID
S Protein + Adjuvant	National Institute of Infectious Disease, Japan/Shionogi/UMN Pharma
VLP-recombinant protein + Adjuvant	Osaka University/BIKEN/National Institutes of Biomedical Innovation, Japan
Microneedle arrays S1 subunit	Univ. of Pittsburgh
Peptide	Vaxil Bio
Adjuvanted protein subunit (RBD)	Biological E Ltd.
Peptide	Flow Pharma Inc
S Protein	AJ Vaccines
Ii-Key peptide	Generex/EpiVax
S Protein	EpiVax/Univ. of Georgia
Protein Subunit EPV-CoV-19	EpiVax
gp-96 backbone	Heat Biologics/Univ. Of Miami
Subunit vaccine	FBRI SRC VB VECTOR, Rospotrebnadzor, Koltsovo
S1 or RBD protein	Baylor College of Medicine
Subunit protein, plant produced	iBio/CC-Pharming
Recombinant protein, nanoparticles (based on S-protein and other epitopes)	Saint-Petersburg scientific research institute of vaccines and serums
COVID-19 XWG-03 truncated S proteins	Innovax/Xiamen Univ./GSK
Adjuvanted microsphere peptide	VIDO-InterVac, University of Saskatchewan
Synthetic Long Peptide Vaccine candidate for S and M proteins	OncoGen
Oral E. coli-based protein expression system of S and N proteins	MIGAL Galilee Research Institute
Nanoparticle vaccine	LakePharma, Inc.,
Plant-based subunit (RBD-Fc + Adjuvant)	Baiya Phytopharm/Chula Vaccine Research Center
OMV-based vaccine	Quadram Institute Biosciences
OMV-based vaccine	BiOMViS Srl/Univ. of Trento
Structurally modified spherical particles of the tobacco mosaic virus (TMV)	Lomonosov Moscow State University
Spike-based	University of Alberta
Recombinant S1-Fc fusion protein	AnyGo Technology
Recombinant protein	Yisheng Biopharma
Recombinant S protein in IC-BEVS (Viral vector vaccine (based on baculovirus expression system in insect cell line)	Vabiotech, Vietnam and University of Bristol, United Kingdom
Orally delivered, heat stable subunit	Applied Biotechnology Institute, Inc.,
Peptides derived from Spike protein	Axon Neuroscience SE
Protein Subunit	MOGAM Institute for Biomedical Research, GC Pharma
RBD-based	Neovii/Tel Aviv University
Outer Membrane Vesicle (OMV)-subunit	Intravacc/Epivax
Spike-based (epitope screening)	ImmunoPrecise/LiteVax BV
Spiked-based	Nanografi Nano Technology, Middle East Technical University, Ankara University
Recombinant spike with adjuvant	Iran
Recombinant S protein produced in BEVS	Tampere University
Protein Subunit Nanoformulated	Vaxinano, CEA, INRAE
Protein Subunit Adenoviral Carrier	CEA, CNRS
Protein DC-targeted epitopes	LinkinVax, VRI

#### Nano Based Therapeutic Approaches

Nanotechnology plays a major role in COVID-19 therapeutics (Petros and DeSimone, 2010). The success of nanotechnology in SARS CoV-2 therapeutics depends on the appropriate choice of nanocarriers for the right drug candidate (Chauhan et al., 2020). Moreover, nanocarriers overcome the limitations of existing antiviral therapies. Nanoparticle aided modulation of antigen presenting cells (APCs) is important for vaccine development in COVID-19 (Banchereau and Steinman 1998; Steinman and Banchereau, 2007). During the initial stages of the COVID-19, nano-macrophage mimetic systems neutralize viral activity and in later stages it reduces the inflammation (Zhang et al., 2020). The effects associated with hematological pathology of COVID-19 can be reduced by Nano-Erythrocyte mimetic drug delivery (Cavezzi et al., 2020; Poduri et al., 2020). Thrombocytopenia and vascular damages induced by COVID-19 can be reduced by Nano-Platelet mimetic drug delivery (Anselmo et al., 2014). The self-amplifying RNA encoding SARS-CoV-2 spike protein was encapsulated in lipid nanoparticles which can act as a vaccine for neutralizing the pseudo-virus (Mckay et al., 2020) (Shin et al., 2019). Layered Double Hydroxide (LDH), an inorganic nanoparticle intercalated with short hairpin RNA (shRNA) plasmid has the potential of gene silencing at the target sequence is employed for SARS-CoV-2. Further, it is formulated as a nasal spray for delivering shRNA at the target sites (Acharya, 2020). The small-interfering RNA (si-RNA) encapsulated in lipidic nano-nanoparticles can be used to inhibit chemokine receptor (CCR2), which is responsible for creating cytokine storms. The cytokine storm is one of the major clinical complications in SARS-CoV-2. The inhibition may result in the reduction of inflammatory sites in the infected regions (Campos et al., 2020). Peptide based vaccines are also developed using Lipid nanoparticle formulation. Table 3 shows the nano-based therapeutic options for Coronavirus.

**TABLE 3:** Nano based therapeutic options for Coronavirus.

Nanoparticle	Coronavirus antigen	Adjuvant	Mechanism	References
Hollow polymeric nanoparticles	MERS-CoV (Middle East Respiratory Syndrome Coronavirus)	STING agonist	Elicits antigen-specific T-cell response without triggering eosinophilic immunopathology	(Lin et al., 2019)
SARS-S, MERS-S (MERS Spike protein nanoparticle)	SARS-CoV, MERS-CoV	Amphiphilic membrane protein aggregates	Production of high levels of neutralizing antibodies in case of homologous viruses and no response against heterologous viruses	(Coleman et al., 2014)
MERS-S	MERS-CoV	Aluminium (alum)	Simultaneous T_H1_ and T_H2_ response in heterologous prime-boost	(Jung et al., 2020)
Gold nanoparticle	SARS-CoV	Gold nanoparticle	Induces strong IgG response	(Sekimukai, Iwata, and Shuetsu, 2020)
Spherical gold nanoparticles	Transmissible Gastroenteritis Virus (TGEV)	Gold nanoparticle	An increased concentration of Interleukins (ILs)	(Staroverov et al., 2019)
Aluminium nanoparticles	SARS-CoV, MERS-CoV	Alum	Increase in number of IFN and immunoglobulins, T_H1_ and T_H2_ Balance	(Wang N. et al., 2020)

**FIGURE 3:**
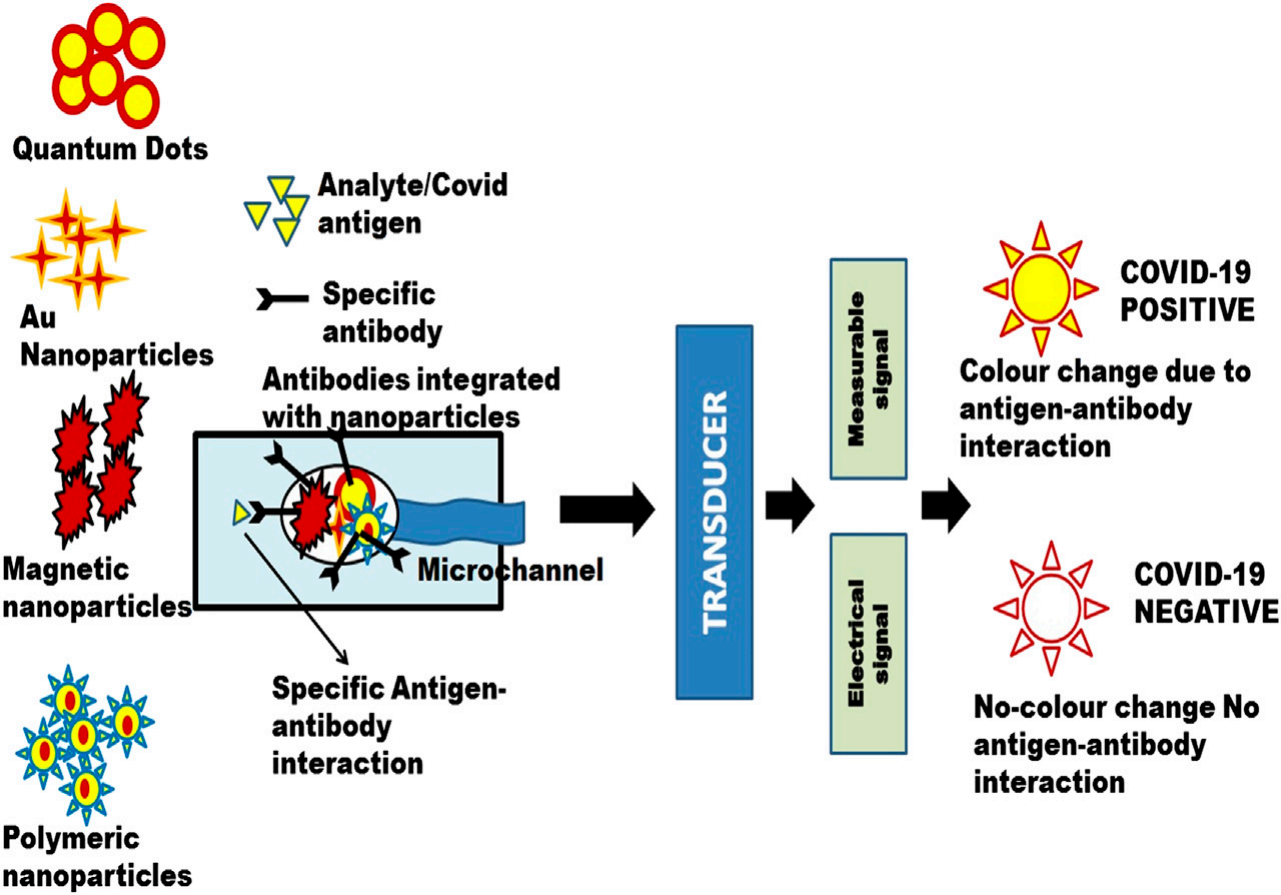
A schematic illustration of nano-based approach to combat Covid-19.

Because of limited side effects, lower dosage quantity and multiple targeting, combination drug therapeutics play a pivotal role in the treatment of COVID-19. Nanocarriers act as potential candidates for multi drug delivery which in turn useful for combination drug therapy (Destache et al., 2009; Shibata et al., 2013). The antigen delivering mode of nanoparticles to dendritic cells facilities T cell immunity (Joffre et al., 2012). Targeted drug delivery for the treatment of COVID-19 can be enhanced by the application of nanomaterials including nanospheres, nanocarriers, liposomes, lipid nanoparticles, nanophages and dendrimers (Witika et al., 2020). Figure 3 depicts the schematic illustration of a nano-based approach to combat COVID-19.

## Present Status and Future Perspectives

Even though the antimicrobial drugs including chloroquine, remdesivir, lopinavir shows promising results against SARS-CoV-2 (Mainardes and Diedrich, 2020), it may cause side effects to some patients. The association of nano-carriers provides a necessary environment for the functioning of these drugs without any harmful effects (Campos et al., 2020). The existing antiviral drugs lack specificity and cause cytotoxicity and which are made to mimic Heparan Sulphate Proteoglycan (HSPG), the first step is virus-cell interaction, which are conserved regions for the ligands of the virus. Unlike invasive carriers, drug delivery of smart nano-carriers is based on the external stimuli like magnetic field or ultrasound (Jindal and Gopinath, 2020). Likewise the effective demonstration of nano-carriers in the treatment of Human Immunodeficiency Virus (HIV), Hepatitis, Influenza A virus etc., (Abd Elkodous et al., 2019; Negahdari et al., 2019; Kim et al., 2020), the existing drugs are being tested for the treatment of SARS-CoV-2 infection. Since SARS-CoV-2 infects the respiratory system, the drugs are administered through non-invasive methods. The aerosols administered without nano-carriers, may not bind with the target. Hence, nano-carrier mediated drug delivery is preferred for the successful binding with the target (Jindal and Gopinath, 2020).

The iron oxide nanoparticles approved by FDA for *in vitro* viral treatment was monitored for the discovery of treatment methods of SARS-CoV-2. The docking studies of iron oxide nanoparticles with the viral S protein of the SARS-CoV-2 exhibited better complex binding, which can be further improved in clinical trial stages (Abo-zeid et al., 2020). Several natural compounds have also been contributed to antiviral therapy. The inhibition of Hepatitis C virus into the hepatoblastoma cells was improved when curcumin loaded chitosan-based nanoparticles are used, it disturbs the viral membrane integrity (Loutfy et al., 2020). The IL-6 and IL-1β mediators of the viral response were diminished when curcumin-based nanoparticles were employed for treating patients with SARS-CoV-2. IL-1β is produced right after viral attachment and IL-6 on the progression of the infection. It is suggested to use nano-curcumin for COVID-19 (Valizadeh et al., 2020).

The application of silver nanoparticles for the treatment of Respiratory Syncytial Virus (RSV) has shown promising results in the mouse model, in which the nanoparticles tend to inhibit the viral replication in the host by binding to the viral glycoprotein and recruiting the neutrophils. Thus, silver nanoparticles reduce the spread of SARS-CoV-2 and are used to prevent the infected patient from ventilator-associated pneumonia (Morris et al., 2019; Issn and Sarkar 2020; Zachar, 2020). The toxicity caused due to the application of chloroquine and hydroxychloroquine for treating SARS-CoV-2 can be reduced by the application of noble nanoparticles (Au, Ag, Pt) (Rezaee et al., 2020). Currently, novel lipid nanoparticle encapsulates mRNA (mRNA-1273) (NCT04283461) is under clinical trial for SARS-CoV-2 by National Institute of Allergy and Infectious Disease (NIAID), United States. It is also suggested that combination of traditional interventions and western medicine for COVID-19 (Ni et al., 2020). China, Japan, South Korea and Singapore got relieved from the spreading because of Traditional Chinese Medicine (TCM) or Chinese Herbal Medicine (CHM) (Xu and Zhang 2020) and death rate is controlled to considerable extent. Peptide vaccines are considered as the most significant therapeutic compounds for viral infections (Agarwal and Gabrani, 2020; Brice and Diamond, 2020; Kalita et al., 2020). Further, novel delivery modes facilitate peptides as prominent vaccine candidates for COVID-19 (Di Natale et al., 2020).

## Conclusion

WHO is kept on track for the research and development of vaccines against SARS-CoV-2 across the world. Out of all the epidemics and pandemics, the vaccine development has been faster for SARS-CoV-2 due to its fast-paced spread throughout the world. Compared to other SARS virus vaccines that have reached clinical trials within 22 months–26 months, SARS-CoV-2 vaccine is the one in history that has reached the clinical trial phases within 3–6 months. But until an effective vaccine is formulated, it is better to control the pandemic using repurposed drugs.
