# Supplementary material for: Emerging Therapeutic Approaches to Combat COVID-19: Present Status and Future Perspectives
Source: Front Mol Biosci. 2021 Mar 8;8:604447. doi: 10.3389/fmolb.2021.604447 (PMC7983051; doi:10.3389/fmolb.2021.604447)
Supplement: Supplementary file 1 [file DataSheet1.docx]

**Appendix**

(WHO - DRAFT landscape of COVID-19 candidate vaccines, December 2020).

| **Vaccine platform description** | **Type of candidate vaccine** | **Developers** |
| --- | --- | --- |
| Inactivated virus | SARS-CoV-2 vaccine (inactivated) | Sinovac Research and Development Co., Ltd |
| Inactivated virus | Inactivated SARS-CoV-2 vaccine (Vero cell) | Sinopharm + Wuhan Institute of Biological Products |
| Inactivated virus | Inactivated SARS-CoV-2 vaccine (Vero cell) | Sinopharm + Beijing Institute of Biological Products |
| Viral vector (Non-replicating) | ChAdOx1-S - (AZD1222) (Covishield) | AstraZeneca + University of Oxford |
| Viral vector (Non-replicating) | Recombinant novel coronavirus vaccine (Adenovirus type 5 vector) | CanSino Biological Inc./Beijing Institute of Biotechnology |
| Viral vector (Non-replicating) | Gam-COVID-Vac Adeno-based (rAd26-S+rAd5-S) | Gamaleya Research Institute ; Health Ministry of the Russian Federation |
| Viral vector (Non-replicating) | Ad26.COV2.S | Janssen Pharmaceutical |
| Protein subunit | SARS-CoV-2 rS/Matrix M1-Adjuvant (Full length recombinant SARS CoV-2 glycoprotein nanoparticle vaccine adjuvanted with Matrix M) | Novavax |
| RNA based vaccine | mRNA -1273 | Moderna + National Institute of Allergy and Infectious Diseases (NIAID) |
| RNA based vaccine | BNT162 (3 LNP-mRNAs ) | BioNTech + Fosun Pharma ; Jiangsu Provincial Center for Disease Prevention and Control + Pfizer |
| Protein subunit | Recombinant SARS-CoV-2 vaccine (CHO Cell) | Anhui Zhifei Longcom Biopharmaceutical + Institute of Microbiology, Chinese Academy of Sciences |
| RNA based vaccine | CVnCoV Vaccine | CureVac AG |
| Inactivated virus | SARS-CoV-2 vaccine (vero cells) | Institute of Medical Biology + Chinese Academy of Medical Sciences |
| Inactivated virus | QazCovid-in® - COVID-19 inactivated vaccine | Research Institute for Biological Safety Problems, Rep of Kazakhstan |
| DNA based vaccine | INO-4800+electroporation | Inovio Pharmaceuticals + International Vaccine Institute |
| DNA based vaccine | AG0301-COVID19 | AnGes + Takara Bio + Osaka University |
| DNA based vaccine | nCov vaccine | Cadila Healthcare Ltd. |
| DNA based vaccine | GX-19 | Genexine Consortium |
| Inactivated virus | Whole-Virion Inactivated SARS-CoV-2 Vaccine (BBV152) | Bharat Biotech International Limited |
| Protein subunit | KBP-COVID-19 (RBD-based) | Kentucky Bioprocessing Inc. |
| Protein subunit | SARS-CoV-2 vaccine formulation 1 with adjuvant 1 (S protein (baculovirus production) | Sanofi Pasteur + GSK |
| RNA based vaccine | ARCT-021 | Arcturus Therapeutics |
| Virus like particle | RBD SARS-CoV-2 HBsAg VLP vaccine | Serum Institute of India + Accelagen Pty |
| Inactivated virus | Inactivated SARS-CoV-2 vaccine (Vero cell) | Shenzhen Kangtai Biological Products Co., Ltd. |
| Viral vector (Non-replicating) | GRAd-COV2 (Replication defective Simian Adenovirus (GRAd) encoding S) | ReiThera + Leukocare + Univercells |
| Viral vector (Non-replicating) | VXA-CoV2-1 Ad5 adjuvanted Oral Vaccine platform | Vaxart |
| Viral vector (Non-replicating) | MVA-SARS-2-S | University of Munich (Ludwig-Maximilians) |
| Protein subunit | SCB-2019 + AS03 or CpG 1018 adjuvant plus Alum adjuvant (Native like Trimeric subunit Spike Protein vaccine) | Clover Biopharmaceuticals Inc./GSK/Dynavax |
| Protein subunit | COVID19 vaccine | Vaxine Pty Ltd. + Medytox |
| Protein subunit | MVC-COV1901 (S-2P protein + CpG 1018) | Medigen Vaccine Biologics + Dynavax + National Institute of Allergy and Infectious Diseases (NIAID) |
| Protein subunit | FINLAY-FR anti-SARS-CoV-2 Vaccine (RBD + adjuvant) | Instituto Finlay de Vacunas |
| Protein subunit | EpiVacCorona (EpiVacCorona vaccine based on peptide antigens for the prevention of COVID-19) | Federal Budgetary Research Institution State Research Center of Virology and Biotechnology "Vector" |
| Protein subunit | RBD (baculovirus production expressed in Sf9 cells) Recombinant SARS-CoV-2 vaccine (Sf9 Cell) | West China Hospital + Sichuan University |
| Protein subunit | IMP CoVac-1 (SARS-CoV-2 HLA-DR peptides) | University Hospital Tuebingen |
| Protein subunit | UB-612 (Multitope peptide based S1-RBD-protein based vaccine) | COVAXX + United Biomedical Inc |
| Viral vector (Replicating) | V591-001 - Measles-vector based (TMV-o38) | Merck & Co. + Themis + Sharp & Dohme + Institute Pasteur + Univeristy of Pittsburgh |
| Viral vector (Replicating) | DelNS1-2019-nCoV-RBD-OPT1 (Intranasal flu-based-RBD ) | Jiangsu Provincial Center for Disease Prevention and Control |
| RNA based vaccine | LNP-nCoVsaRNA | Imperial College London |
| RNA based vaccine | SARS-CoV-2 mRNA vaccine | Shulan (Hangzhou) Hospital + Center for Disease Control and Prevention of Guangxi Zhuang Autonomous Region |
| Virus like particle | Coronavirus-Like Particle COVID-19 (CoVLP) | Medicago Inc. |
| Viral vector (Replicating) + APC | Covid-19/aAPC vaccine. The Covid-19/aAPC vaccine is prepared by applying lentivirus modification with immune modulatory genes and the viral minigenes to the artificial antigen presenting cells (aAPCs). | Shenzhen Geno-Immune Medical Institute |
| Viral vector (Non-replicating) + APC | LV-SMENP-DC vaccine. Dendritic cells are modified with lentivirus vectors expressing Covid-19 minigene SMENP and immune modulatory genes. CTLs are activated by LV-DC presenting Covid-19 specific antigens. | Shenzhen Geno-Immune Medical Institute |
| Protein subunit | AdimrSC-2f (recombinant RBD +/- Aluminium) | Adimmune Corporation |
| DNA based vaccine | Covigenix VAX-001 | Entos Pharmaceuticals Inc. |
| DNA based vaccine | CORVax | Providence Health & Services |
| RNA based vaccine | ChulaCov19 mRNA vaccine | Chulalongkorn University |
| DNA based vaccine | bacTRL-Spike | Symvivo Corporation |
| Viral vector (Non-replicating) | hAd5-S-Fusion+N-ETSD vaccine | ImmunityBio, Inc. |
| Viral vector (Non-replicating) | COH04S1 (MVA-SARS-2-S) | City of Hope Medical Center + National Cancer Institute |
| Viral vector (Replicating) | rVSV-SARS-CoV-2-S Vaccine | Israel Institute for Biological Research |
| Viral vector (Replicating) + APC | Dendritic cell vaccine AV-COVID-19. A vaccine consisting of autologous dendritic cells loaded with antigens from SARS-CoV-2, with or without GM-CSF | Aivita Biomedical, Inc. |
| Live attenuated virus | COVI-VAC | Codagenix/Serum Institute of India |
| Protein subunit | CIGB-669 (RBD+AgnHB) | Center for Genetic Engineering and Biotechnology (CIGB) |
| Protein subunit | CIGB-66 (RBD+aluminium hydroxide) | Center for Genetic Engineering and Biotechnology (CIGB) |
| Inactivated Virus | VLA2001 | Valneva, National Institute for Health Research, United Kingdom |
| Protein subunit | BECOV2 | Biological ELimited |
| Viral vector (Replicating) | AdCLD-CoV19 | Cellid Co., Ltd. |
| DNA based vaccine | GLS-5310 | GeneOne Life Science, Inc. |
| Protein subunit | Recombinant Sars-CoV-2 Spike protein, Aluminum adjuvanted | Nanogen Pharmaceutical Biotechnology |
| Protein subunit | Recombinant protein vaccine S-268019 (using Baculovirus expression vector system) | Shionogi |
